# Relationship of Serum 3-Nitrotyrosine Levels with Inflammation in Patients with Rheumatoid Arthritis

**DOI:** 10.3390/diagnostics15111325

**Published:** 2025-05-25

**Authors:** Juan C. Quevedo-Abeledo, Fuensanta Gómez-Bernal, María García-González, Marta Hernández-Díaz, Cristina Almeida-Santiago, Pedro Abreu-González, Candelaria Martín-González, Miguel Á. González-Gay, Iván Ferraz-Amaro

**Affiliations:** 1 Division of Rheumatology, Hospital Doctor Negrín, 35010 Las Palmas de Gran Canaria, Spain; quevedojcarlos@yahoo.es (J.C.Q.-A.); almeidasantiago.cristina@gmail.com (C.A.-S.); 2 Division of Central Laboratory, Hospital Universitario de Canarias, 38320 Tenerife, Spain; fuensanta95@gmail.com; 3 Division of Rheumatology, Hospital Universitario de Canarias, 38320 Tenerife, Spain; margagon23@hotmail.com (M.G.-G.); martahediaz@gmail.com (M.H.-D.); 4 Unit of Physiology, Department of Basic Medical Sciences, University of La Laguna, 38200 Tenerife, Spain; pabreu@ull.edu.es; 5 Department of Internal Medicine, University of La Laguna (ULL), 38200 Tenerife, Spain; mmartgon@ull.edu.es; 6 Division of Rheumatology, IIS-Fundación Jiménez Díaz, 28040 Madrid, Spain; 7 Department of Medicine and Psychiatry, University of Cantabria, 39005 Santander, Spain

**Keywords:** 3-nitrotyrosine, rheumatoid arthritis, cardiovascular disease

## Abstract

**Objective:** 3-Nitrotyrosine (3-NT) is a byproduct of tyrosine nitration, mediated by reactive nitrogen species such as peroxynitrite and nitrogen dioxide. It serves as a marker of cellular damage, inflammation, and nitric oxide activity. Rheumatoid arthritis (RA) is a complex autoimmune disease characterized by systemic involvement and increased oxidative stress. In RA patients, cardiovascular disease has emerged as the leading cause of mortality. This study aimed to investigate the relationship between serum 3-NT levels and various disease characteristics in RA patients, with a particular focus on cardiovascular comorbidities. **Methods:** A total of 168 RA patients were recruited. They underwent comprehensive evaluations, including disease-related characteristics and disease activity indices. Furthermore, a comprehensive lipid panel, measures of insulin resistance, metabolic syndrome criteria, and carotid ultrasound to evaluate intima–media thickness and the presence of carotid plaques were conducted. 3-NT serum levels were measured. A multivariable linear regression analysis was performed to examine the associations between the disease characteristics and 3-NT. **Results:** After multivariable analysis, C-reactive protein was independently associated with higher serum levels of 3-NT. In contrast, disease characteristics and Disease Activity Score 28-joint count (DAS28) calculated using C-reactive protein or erythrocyte sedimentation rate, showed no significant association with 3-NT levels. Likewise, cardiovascular comorbidities, including lipid profiles, insulin resistance indices, metabolic syndrome, and markers of subclinical atherosclerosis did not demonstrate any significant relationship with 3-NT levels. **Conclusions:** While 3-NT levels are influenced by inflammation, they do not appear to be strongly associated with disease characteristics, cardiovascular risk, or disease-modifying anti-rheumatic drugs in RA patients. This emphasizes the complexity of oxidative stress in RA.

## 1. Introduction

Rheumatoid arthritis (RA) is a long-lasting inflammatory condition of unknown cause, mainly identified by symmetrical inflammation of multiple joints in the extremities. It can also affect different organs and tissues beyond the joints, resulting in skin nodules, eye problems like dryness, episcleritis, and scleritis, lung disease, blood abnormalities, and neurological complications [1]. Patients with RA exhibit an elevated risk of cardiovascular (CV) events, including myocardial infarction and CV-related mortality, when compared to the general population [2,3]. Insulin resistance [4,5] and metabolic syndrome, which is characterized by the simultaneous presence of abdominal obesity, hyperglycemia, dyslipidemia, and hypertension, are frequently observed in patients with RA [6,7].

Oxidative stress has been shown to play a role in the initiation and progression of RA. The exact mechanisms by which oxidative stress contributes to the onset and propagation of local joint inflammation and systemic involvement in RA remain to be fully elucidated. However, environmental pollution, tobacco use, diet, and imbalances in microbiota have been proposed as potential sources of increased reactive oxygen species production in RA [8]. Nitrosative stress and lipid peroxidation may contribute to DNA, lipid, and protein damage, thereby promoting synovial inflammation and joint destruction in RA [9].

3-nitrotyrosine (3-NT) is a post-translational protein modification formed by the nitration of tyrosine residues, a process involving the addition of a nitro (-NO2) group by nitrating agents. [10]. By potentially altering protein structure and function, this modification, which is recognized as a specific marker of peroxynitrite-mediated oxidative damage, may contribute to cellular dysfunction in various pathological conditions. In this context, the presence of 3-NT has been documented across numerous disease states [11]. For example, elevated levels of 3-NT have been reported is various human pathologies such as atherosclerosis and CV disease [12], neurodegenerative diseases like multiple sclerosis, Alzheimer’s or Parkinson’s diseases [13] and lung disorders [14]. It is hypothesized that peroxynitrite-mediated oxidation and nitration of biomolecules contribute to the development of autoimmune diseases. When these modified proteins are released, they may trigger an immune response, leading to autoantibody production. Tyrosine-nitrated proteins, functioning as neoantigens, have the potential to induce the generation of autoantibodies against native proteins in various autoimmune disorders [10]. This mechanism suggests a connection between oxidative stress, protein modification, and the loss of immune tolerance in autoimmune pathogenesis.

Previously, no studies have examined the association between circulating 3-NT levels and disease characteristics in RA patients. In this study, we measured serum 3-NT levels in a large cohort of RA patients, who were comprehensively characterized not only by disease-related features but also by CV comorbidities. We then analyzed the relationship between serum 3-NT levels and these characteristics. Understanding the relationship between 3-NT and inflammation in RA may provide further insights into the disease’s pathophysiology and potentially inform future therapeutic strategies

## 2. Materials and Methods

### 2.1. Study Participants

This was a cross-sectional study that included 168 consecutively recruited RA patients, all of whom were 18 years or older and met the 2010 American Colleague of Rheumatology and European Alliance of Associations for Rheumatology—ACR/EULAR—classification criteria [15]. They had been diagnosed by rheumatologists and attended regular follow-up appointments at rheumatology outpatient clinics. For the purpose of inclusion in the present study, the duration of RA disease was required to be ≥1 year. As glucocorticoids are frequently employed in the management of RA, patients receiving prednisone or an equipotent dose of ≤10 mg/daily were enrolled in the study. Individuals with a history of malignancy, chronic comorbidities (e.g., hypothyroidism, CV or respiratory disease, and nephrotic syndrome), or active infectious processes were excluded. The study protocol received ethical approval from the Institutional Review Boards at Hospital Universitario de Canarias and Hospital Universitario Doctor Negrín (approval number 2023-48), and all participants provided written informed consent prior to enrollment. The research was conducted in compliance with relevant ethical guidelines and the Declaration of Helsinki.

### 2.2. Data Collection

Participants were subjected to a thorough clinical evaluation, encompassing a structured questionnaire eliciting data on CV risk factors and current medication usage. The physical examination involved the assessment of body mass index (BMI), waist circumference, and blood pressure (both systolic and diastolic readings) under standardized measurement protocols. Data regarding smoking status, the presence of diabetes mellitus, and hypertension were collected, and specific medical diagnoses and medication regimens were verified through review of electronic health records. Disease activity in patients with RA was measured using the Disease Activity Score (DAS28) in 28 joints [16], the Clinical Disease Activity Index (CDAI) [17], and the Simple Disease Activity Index (SDAI) [18]. DAS28-ESR (erythrocyte sedimentation rate) and DAS28-CRP (C-reactive protein) were classified into distinct categories based on predefined thresholds: remission (<2.6), low (>2.6 to 3.2), moderate (>3.2 to 5.1), or high disease activity (>5.1) as previously described [19]. Likewise, the SDAI categories were defined as follows: remission (≤3.3), low disease activity (>3.3 and ≤11), moderate disease activity (>11 and ≤26), and high disease activity (>26). Similarly, the CDAI was categorized into remission (≤2.8), low disease activity (>2.8 and ≤10), moderate disease activity (>10 and ≤22), and high disease activity (>22). These categorizations adhere to established criteria [20].

The presence of metabolic syndrome was determined using the National Cholesterol Education Program (NCEP/ATPIII) criteria [21]. Based on these criteria, metabolic syndrome is diagnosed upon the presence of three or more of the following five parameters: abdominal obesity, defined as a waist circumference exceeding 102 cm in males or 88 cm in females; elevated blood pressure, defined as a systolic blood pressure ≥ 130 mmHg or a diastolic blood pressure ≥ 85 mmHg; hypertriglyceridemia, defined as a fasting triglyceride level ≥ 150 mg/dL; low high-density lipoprotein (HDL) cholesterol, defined as a fasting HDL cholesterol level < 40 mg/dL in males or <50 mg/dL in females; and elevated fasting glucose, defined as a fasting blood glucose level ≥ 100 mg/dL [21]. The Systematic Coronary Risk Evaluation-2 (SCORE2) CV risk tool was calculated as previously described using age, gender, smoking status, systolic blood pressure, and non-HDL-cholesterol [22]. The SCORE2 algorithm calculates the 10-year probability of experiencing fatal and non-fatal CV disease events in individuals aged 40 to 69 years. For apparently healthy individuals aged 70 years and older, the SCORE2-Older Persons (SCORE2-OP) algorithm estimates the 5-year and 10-year risk of fatal and non-fatal CV disease events.

### 2.3. Laboratory Assessments

Serum samples were collected from patients and immediately cryopreserved at −80 °C until analysis. Serum cholesterol, triglycerides, and high-density lipoprotein cholesterol (HDL-C) were quantified using enzymatic colorimetric methods with reagents from Roche Farma (Madrid, Spain). Lipoprotein levels in serum were determined using a quantitative immunoturbidimetric assay also from Roche Farma (Madrid, Spain). The measured ranges and intra-assay coefficients of variation were as follows: cholesterol, 0.08–20.7 mmol/L (CV = 0.3%); triglycerides, 4–1000 mg/dL (CV = 1.8%); and HDL-C, 3–120 mg/dL (CV = 0.9%). The atherogenic index was calculated as the ratio of total cholesterol to HDL-C, based on the Castelli formula. Low-density lipoprotein (LDL) cholesterol was calculated using the Friedewald formula (LDL = cholesterol − HDL − (triglycerides/5) [23]. The erythrocyte sedimentation rate (ESR) was determined using the Westergren method. This method involves measuring the distance in millimeters that red blood cells settle in a vertical glass tube over one hour [24]. High-sensitivity C-reactive protein (hs-CRP) levels were measured using a high-sensitivity nephelometry immunoassay.

The 3NT concentration in serum was evaluated using a solid-phase competitive Enzyme Linked Immunosorbent Assay (Invitrogen, Thermo Fisher Scientific Inc., Whaltam, MA, USA), according to the manufacturer’s protocol. Briefly, in each appropriate well, 50 μL of standards, 50 μL of serum without dilution, and 50 μL of Biotinylated Detection Ab working solution were incubated for 45 min at 37 °C. After washing each well with wash buffer, 100 μL of HRP Conjugate working solution was added into each well and incubated for 30 min at 37 °C. After washing each well with wash buffer, 90 μL of substrate reagent (3,3′,5,5′-Tetramethylbenzidine or TMB as a chromogenic substrate) was added to each well and incubated for 15 min at 37 °C, avoiding direct light. Finally, 50 μL of stop solution (1 M H_2_SO_4_) was added to each well (yellow color) at room temperature. The kit uses a calibration curve with a range of 0–100 ng/mL. Samples and standard absorbance units were read in a microplate spectrophotometer at 450 nm (Spectra MAX-190, Molecular Devices, Sunnyvale, CA, USA). A four-parameter algorithm provided the best standard curve fit (the correlation coefficients, r2, of different kits ranged between 0.9932 and 0.9967). The limit of detection (LOD) of the different assays was established at 0.64 ng/mL. Intra-assay and inter-assay coefficients of variation (CVs) were calculated at 5.21% and 5.43%, respectively.

The homeostatic model assessment (HOMA) method was performed to determine IR. Briefly, the HOMA model enabled an estimate of insulin sensitivity (%S) and β-cell function (%B) from fasting plasma insulin, C-peptide, and glucose concentrations. In this investigation, we employed HOMA2, the modernized computerized HOMA model [25]. This analytical tool enables assessment of insulin sensitivity and beta-cell function using paired fasting measurements of plasma glucose and either specific insulin or C-peptide concentrations, functioning across ranges of 1–2200 pmol/L for insulin and 1–25 mmol/L for glucose. Since C-peptide serves as a secretion marker, it provides superior estimates of beta-cell function; conversely, insulin measurements are preferred for %S calculations as HOMA-%S reflects glucose disposal relative to insulin concentration. For our analysis, insulin serum levels were utilized to determine IR and %S, while C-peptide serum levels were used for %B calculations. The computerized model generated insulin sensitivity values expressed as HOMA2-%S (where 100% represents normal sensitivity). The insulin resistance index (HOMA2-IR) simply represents the reciprocal of %S. We measured insulin using chemiluminescent immunometric assays (Architect Abbott, 2000I, Madrid, Spain) and C-peptide (Immulite 2000, Siemens, Munich, Germany) through the same methodology. The chemiluminescent immunometric assay is a highly sensitive technique where an antibody labeled with a chemiluminescent substance binds to the target molecule. The resulting immune complex emits light through a chemical reaction, and the intensity of this light is measured to quantify the target molecule’s concentration.

### 2.4. Carotid Ultrasound Assessment

Carotid intima–media thickness (cIMT) of the common carotid artery was evaluated via carotid ultrasonography to detect any localized plaques within the extracranial carotid arteries. The assessment was performed using the EsaoteMylab 70 ultrasound system (Genova, Italy), which incorporates a 7–12 MHz linear transducer and the Esaote-developed Quality Intima Media Thickness (QIMT), a real-time automated radiofrequency technique from Maastricht, Netherlands. The assessment process followed the guidelines established by the Mannheim Consensus [26], which establishes criteria for identifying plaques within the accessible extracranial carotid arteries. Plaque criteria were established as the presence of a localized bulge within the arterial lumen, with a measurement of cIMT exceeding >1.5 mm. Additionally, the bulge needed to be at least 50% larger than the adjacent cIMT or result in an arterial lumen reduction of >0.5 mm [26].

### 2.5. Statistical Analysis

The demographic and clinical features of the RA patient cohort were described as means with standard deviations for continuous variables following a normal distribution, and as percentages for categorical variables. For continuous variables not normally distributed, medians and interquartile ranges (IQRs) were reported. The association between disease characteristics and 3-nitrotyrosine (3-NT) levels was evaluated using multivariable linear regression models, with adjustment for covariates demonstrating an association with 3-NT at a significance level of *p* < 0.20. All analyses were performed at a 5% two-sided significance level using Stata software, version 17/BE (StataCorp, College Station, TX, USA). *p*-values < 0.05 were considered statistically significant.

## 3. Results

### 3.1. Demographics and Disease-Related Data

This study included a total of 168 patients diagnosed with RA. The 3-NT values in our study were 43 ± 29 ng/mL. Demographic- and disease-related characteristics of the participants are shown in Table 1. The average age within the study population was 58 ± 9 years, and women comprised 85% of the cohort. The mean BMI was 30 ± 5 kg/m^2^. Traditional CV risk factors were common: 19% of participants were current smokers, 15% had diabetes mellitus, and 42% were diagnosed with hypertension. Additionally, metabolic syndrome was present in 59% of the group, while 42% and 12% were on statin and aspirin therapy, respectively. The median SCORE2 CV risk score was 4.6 (IQR 2.3–6.0), categorizing 58% of patients as low risk, 34% as intermediate risk, and 8% as high risk. For subclinical atherosclerosis, the mean cIMT measured 715 ± 148 microns, with carotid plaques detected in 52% of patients. Comprehensive information on the lipid profile and insulin resistance indices can be found in Table 1.

Disease duration in the cohort had a median of 13 years (IQR 8–185). The inflammatory markers at study assessment showed mean hs-CRP levels of 2.0 mg/L (IQR 1.0–4.4) and ESR values of 16 mm/1st hour (IQR 8–26). Seropositivity was common, with rheumatoid factor detected in 83% of patients and anti-citrullinated protein antibodies (ACPAs) present in 74%. Regarding disease activity indices, the DAS28-ESR averaged 2.85 ± 1.11, while DAS28-CRP was 2.45 ± 0.92. The median SDAI and CDAI scores were 12 (IQR 6–21) and 7 (IQR 3–15), respectively. Current treatment included prednisone in 34% of patients, and 86% were on at least one conventional disease-modifying anti-rheumatic drug (DMARD), with methotrexate being the predominant choice (64%).

Twenty-one percent of the patients were receiving anti-tumor necrosis factor therapies. The frequency of use of other treatments and historical disease-related data are provided in Table 1.

### 3.2. Analysis of the Relationship Between 3-NT and Cardiovascular Traditional Risk Factors, Lipid Pattern, Insulin Resistance and Carotid Subclinical Atherosclerosis

Regarding demographic characteristics, only female sex was significantly positively associated with 3-NT levels (Table 2). Age, anthropometric variables such as BMI and waist or abdominal circumference, and the presence of CV risk factors or metabolic syndrome did not exhibit significant associations with 3-NT. Additionally, the SCORE2 CV risk calculator showed no significant relationship with 3-NT levels. Similarly, no significant associations were found between 3-NT levels and insulin resistance indices or lipid profile parameters, except for the ApoB/ApoA1 ratio, which exhibited a positive association with serum 3-NT levels after multivariate adjustment. Furthermore, cIMT and the presence of carotid plaque showed no significant correlations with 3-NT concentrations (Table 2).

### 3.3. Analysis of the Association of Disease-Related Data with 3-NT

The multivariate relationship between disease characteristics and 3-NT is presented in Table 3. These analyses were adjusted for age and sex where applicable. No significant associations were observed between serum 3-NT levels and disease characteristics, including disease duration, rheumatoid factor or ACPA status, and disease activity measured using the DAS28-ESR and DAS28-CRP indices. Only CRP and the SDAI—which incorporates CRP—demonstrated significant positive correlations with 3-NT levels after multivariable adjustment. Additionally, serum 3-NT concentrations showed no association with the use of various DMARD therapies.

## 4. Discussion

In this study, we explored the relationship between serum 3-NT levels and various disease characteristics in RA patients, with a focus on CV comorbidities and treatment factors. To our knowledge, our study is the first to measure 3-NT levels in a large and well-characterized cohort of RA patients. Our analysis revealed a few key insights. In this regard, we observed that female sex was significantly associated with higher 3-NT levels. This may suggest a gender-related difference in oxidative stress or inflammatory processes, which could be influenced by hormonal or genetic factors that warrant further investigation. Despite examining multiple disease-related features, including disease duration and disease activity indices such as the DAS28-ESR and DAS28-CRP, we found no significant associations with serum 3-NT levels. This indicates that the presence of general disease activity or specific autoimmune markers does not directly correlate with 3-NT levels in this cohort. However, both CRP and the SDAI were positively correlated with 3-NT levels after multivariate adjustment. This is consistent with the known role of CRP as a marker of inflammation. The difference in the correlation between 3-NT levels and SDAI, but not with DAS28-ESR and DAS28-CRP, may stem from the fact that SDAI includes CRP, a marker more directly related to systemic inflammation, while DAS28-ESR and DAS28-CRP focus more on joint-specific disease activity, making them less sensitive to 3-NT, which may reflect broader inflammatory processes.

Despite the potential association of 3-NT with systemic inflammation, in our study, SCORE2, the CV risk calculator, showed no significant correlation with 3-NT levels. This suggests that while RA patients often have elevated oxidative stress, the direct link between 3-NT and CV risk, as assessed using SCORE2, might not be as prominent. Additionally, we observed no significant correlation between 3-NT and markers like cIMT or the presence of carotid plaques, which are indicators of subclinical atherosclerosis. This may imply that while oxidative stress is present, other factors may drive CV risk in RA, and 3-NT alone may not fully capture this risk.

We also investigated the potential impact of DMARDs on 3-NT levels. Interestingly, serum 3-NT concentrations showed no significant association with the use of various DMARDs, including methotrexate and biologic agents. This suggests that, in this cohort, the treatment regimen did not have an effect on oxidative stress as measured by 3-NT. It could be that the anti-inflammatory effects of these drugs do not directly influence the nitrosative stress pathway.

To the best of our knowledge, no studies have examined the association between circulating 3-NT levels and disease characteristics in RA patients. However, some research has investigated its expression in synovial tissue or fluid. In a previous study involving twenty-two patients with active RA, six with early RA, six healthy controls, and three with osteoarthritis, 3-NT was detected in the serum and synovial fluid of RA patients but not in the control subjects. Notably, early RA patients exhibited significantly lower 3-NT levels compared to those with established RA [27]. Similarly, in the inflamed synovium, 3-NT has been detected in the vascular smooth muscle and macrophages [28]. In addition, antibodies recognizing 3-NT have been identified in the synovium of RA patients [29]. Furthermore, a previous study involving 18 patients with RA found elevated plasma 3-NT levels compared to healthy controls [30]. Notably, a reduction in plasma 3-NT was observed following 6 months of anti-tumor necrosis factor therapy [30]. In keeping with our study, the previously mentioned research on synovial fluid or tissue failed to establish correlations between 3-NT and disease activity, RF or ACPA status, or treatment regimens. These findings suggest that 3-NT, as a marker of protein oxidative stress, does not have significant relevance as a biomarker for clinical disease expression in RA.

As mentioned above, we found a significant positive relationship between 3-NT and CRP that persisted after multivariable adjustment. This finding aligns with a previous study in which plasma 3-NT positively correlated with CRP in 18 RA patients [30]. This positive association suggests that oxidative stress, as reflected by increased 3-NT levels, may be linked to systemic inflammation, especially as mediated by CRP. The relationship between CRP and 3-NT could also indicate that inflammation drives the production of reactive nitrogen species, contributing to joint damage and systemic involvement in RA.

It has been previously demonstrated that the formation of 3-NT on isolated low-density lipoproteins can promote the formation and uptake of foam cells into the blood vessel wall in RA patients [31]. According to that, 3-NT may represent a specific marker of interest when linking oxidative stress with CV disease risk in RA, a disease associated with accelerated atherosclerosis. In this regard, during atherosclerosis, foam cells accumulate in the subendothelial space of lesions, creating a chronic inflammatory environment that releases reactive oxygen and nitrogen species. These reactive species can nitrify tyrosine residues in proteins, forming 3-NT. Elevated levels of 3-NT-modified proteins have been detected in both plasma and atherosclerotic lesions of patients, suggesting a significant association between 3-NT levels and CV risk [32]. Similarly, 3-NT levels are associated with the presence of coronary artery disease and seem to decrease with statin therapy [33]. However, in our study, we did not find an association between 3-NT levels and a range of CV comorbidities in RA patients, including lipid profile, insulin resistance, subclinical carotid atheromatosis, statin use, or the SCORE2 calculator. Considering these findings, it is possible that in RA, the mechanisms driving the increased CV risk may be more complex and not specifically mediated by 3-NT levels.

Regarding other immune-mediated diseases, 3-NT has also been investigated in conditions such as systemic lupus erythematosus and scleroderma. For example, elevated serum levels of 3-NT have been observed in systemic sclerosis patients compared to healthy controls [34]. On the other hand, multiple studies have demonstrated elevated 3-NT levels in systemic lupus erythematosus patients compared to controls [35,36,37,38,39,40]. In this disorder, higher serum 3-NT correlated with active lupus nephritis [35], high SLEDAI disease activity scores or anti-dsDNA antibody levels [38], and renal involvement items within the SLEDAI disease activity score [36]. These findings underscore the role that oxidative stress, and 3-NT specifically, may play in autoimmune diseases.

We acknowledge that we did not include a control group. However, the purpose of our study was not to compare 3-NT levels between controls and RA patients, but rather to explore the relationship between this molecule and disease characteristics. Furthermore, the cross-sectional nature of our work prevents inferring causality, for which prospective studies would be required.

## 5. Conclusions

In conclusion, our findings suggest that while 3-NT levels are influenced by inflammation, as indicated by correlations with CRP and the SDAI, they do not appear to be strongly associated with disease characteristics, CV risk, or DMARD usage in RA patients. This emphasizes the complexity of oxidative stress in RA, highlighting the need for further research to better understand the mechanisms underlying these associations.

## Figures and Tables

**Table 1:** Demographics, cardiovascular risk factors, and disease-related data in RA patients.

	Rheumatoid Arthritis	
	(n = 168)	References Ranges
3-nitrotyrosine, ng/mL	43 ± 29	
Age, years	58 ± 9	
Female, n (%)	142 (85)	
BMI, kg/m^2^	30 ± 5	18.5–24.9
Abdominal circumference, cm	97 ± 13	<80 W, <90 M
Hip circumference, cm	104 ± 11	
Abdominal to hip ratio	0.93 ± 0.08	<0.90
Cardiovascular data		
CV risk factors, n (%)		
Current smoker	32 (19)	
Obesity	74 (44)	
Hypertension	71 (42)	
Diabetes Mellitus	25 (15)	
Metabolic syndrome, n (%)	98 (59)	
Statins, n (%)	70 (42)	
Aspirin, n (%)	20 (12)	
SCORE2, %	4.6 (2.3–6.0)	
Low to moderate	97 (58)	
High	57 (34)	
Very high	14 (8)	
Carotid ultrasound		
cIMT, microns	715 ± 148	
Carotid plaque, n (%)	88 (52)	
Lipid profile		
Total cholesterol, mg/dL	189 ± 40	<200
Triglycerides, mg/dL	145 ± 66	<150
HDL-cholesterol, mg/dL	55 ± 15	>50 W, >40 M
LDL-cholesterol, mg/dL	105 ± 34	<100
LDL:HDL cholesterol ratio	2.06 ± 0.88	<3.5
Non-HDL cholesterol, mg/dL	134 ± 37	<130
Lipoprotein (a), mg/dL	28 (10–92)	<30
Apolipoprotein A1, mg/dL	164 ± 31	130–175 W, 120–160 M
Apolipoprotein B, mg/dL	93 ± 25	90–110
Apo B:Apo A1 ratio	0.60 ± 0.28	<0.9
Atherogenic index	3.68 ± 1.19	<4
Insulin resistance indices		
Glucose, mg/dL	95 ± 28	70–110
Insulin, µU/mL	12.0 (7.3–23.2)	2–25
C-peptide, ng/mL	3.92 ± 2.59	0.8–3.9
HOMA2-IR	1.6 (0.9–2.8)	<1.0
HOMA2-S%	77 ± 52	>100
HOMA2-B%-C-peptide	182 ± 74	≈100
Disease related data		
Disease duration, years	13 (8–18)	
CRP at time of study, mg/L	2.0 (1.0–4.4)	1.0–3.0
ESR at time of study, mm/1° hour	16 (8–26)	0–20
Rheumatoid factor, n (%)	138 (83)	
ACPA, n (%)	122 (74)	
DAS28-ESR	2.85 ± 1.11	
Remission	74 (46)	
Low disease activity	35 (21)	
Moderate disease activity	52 (31)	
High disease activity	5 (3)	
DAS28-CRP	2.45 ± 0.92	
Remission	98 (61)	
Low disease activity	31 (19)	
Moderate disease activity	32 (20	
High disease activity	0 (0)	
SDAI	12 (6–21)	
Remission	17 (11)	
Low disease activity	58 (36)	
Moderate disease activity	57 (35)	
High disease activity	29 (18)	
CDAI	7 (3–15)	
Remission	42 (25)	
Low disease activity	65 (39)	
Moderate disease activity	42 (25)	
High disease activity	19 (11)	
History of extraarticular manifestations, n (%)	45 (27)	
Erosions, n (%)	75 (47)	
Current drugs, n (%)		
Prednisone	57 (34)	
Prednisone doses, mg/day	5 (2.5–5)	
NSAIDs	62 (37)	
DMARDs	145 (86)	
Methotrexate	108 (64)	
Leflunomide	37 (22)	
Anti TNF therapy	36 (21)	
Tocilizumab	7 (4)	
Rituximab	10 (6)	
Abatacept	10 (6)	
Baricitinib	3 (2)	
Tofacitinib	3 (2)	

Data represent means ± SDs or medians (IQRs) when data were not normally distributed. CV: cardiovascular; LDL: low-density lipoprotein; HDL: high-density lipoprotein; CRP: C-reactive protein; NSAID: nonsteroidal anti-inflammatory drugs; DMARD: disease-modifying antirheumatic drug; TNF: tumor necrosis factor; ESR: erythrocyte sedimentation rate; BMI: body mass index; DAS28: Disease Activity Score in 28 joints; ACPA: anti-citrullinated protein antibody; HOMA: homeostatic model assessment; CDAI: Clinical Disease Activity Index; SDAI: Simple Disease Activity Index; cIMT: carotid intima—media thickness; SCORE2: Systematic Coronary Risk Evaluation-2; W: women; M: men.

**Table 2:** Cardiovascular disease-related data association with 3-nitrotyrosine.

	3-Nitrotyrosine, ng/mL			
	Univariable		Multivariable	
	Beta Coefficient (Confidence Interval 95%), *p*			
Age, years	−0.5 (−1–0.7)	0.087		
Female	**14 (0.4–27)**	**0.044**		
BMI, kg/m^2^	−0.1 (−1–0.8)	0.81		
Abdominal circumference, cm	−0.2 (−0.6–0.2)	0.31		
Hip circumference, cm	−0.1 (−0.6–0.3)	0.63		
Abdominal to hip ratio	−36 (−99–26)	0.25		
Cardiovascular data				
CV risk factors				
Current smoker	4 (−8–16)	0.51		
Obesity	3 (−7–12)	0.58		
Hypertension	−4 (−13–6)	0.48		
Diabetes Mellitus	−3 (−17–12)	0.73		
Metabolic syndrome	−6 (−15–4)	0.26		
Statins	−9 (−19–0.7)	0.068	−6 (−16–4)	0.22
Aspirin	−3 (−18–13)	0.74		
SCORE2, %	−1 (−3–0.4)	0.16		
Low to moderate	ref.			
High	2 (−9–12)	0.74		
Very high	8 (−13–29)	0.46		
Lipid profile				
Total cholesterol, mg/dL	−0.05 (−0.2–0.07)	0.42		
Triglycerides, mg/dL	−0.03 (−0.1–0.05)	0.43		
HDL-cholesterol, mg/dL	−0.01 (−0.3–0.3)	0.95		
LDL-cholesterol, mg/dL	−0.05 (−0.2–0.09)	0.50		
LDL:HDL cholesterol ratio	−0.02 (−5–5)	0.99		
Non-HDL cholesterol, mg/dL	−0.05 (−0.2–0.07)	0.40		
Lipoprotein (a), mg/dL	−0.02 (−0.08–0.05)	0.64		
Apolipoprotein A1, mg/dL	−0.03 (−0.2–0.1)	0.73		
Apolipoprotein B, mg/dL	−0.1 (−0.3–0.1)	0.36		
Apo B:Apo A1 ratio	14 (−2–30)	0.085	**20 (4–37)**	**0.016**
Atherogenic index	1 (−2–5)	0.44		
Insulin resistance indices				
Glucose, mg/dL	−0.2 (−0.3–0.02)	0.083	−0.1 (−0.3–0.07)	0.22
Insulin, µU/mL	−0.07 (−0.2–0.1)	0.44		
C-peptide, ng/mL	−1 (−3–0.6)	0.18	−0.9 (−3–1)	0.34
HOMA2-IR	−2 (−4–0.9)	0.22		
HOMA2-S%	0.05 (−0.04–0.1)	0.28		
HOMA2-B%-C-peptide	0.01 (−0.05–0.08)	0.66		
Carotid ultrasound				
cIMT, microns	−0.02 (−0.05–0.02)	0.33		
Carotid plaque	−6 (−15–4)	0.26		

In this analysis, 3-NT is the dependent variable. Multivariable analysis is adjusted for sex and age. CV: cardiovascular; LDL: low-density lipoprotein; HDL: high-density lipoprotein; BMI: body mass index; cIMT: carotid intima–media thickness; HOMA: homeostatic model assessment; SCORE2: Systematic Coronary Risk Evaluation-2. Significant *p* values are depicted in bold.

**Table 3:** Disease-related data association with 3-nitrotyrosine.

	3-Nitrotyrosine, ng/mL			
	Univariable		Multivariable	
	Beta Coefficient (Confidence Interval 95%), *p*			
Disease duration, years	−0.2 (−0.8–0.3)	0.41		
CRP at time of study, mg/L	**0.4 (0.1–0.6)**	**0.006**	**0.5 (0.2–0.7)**	**0.001**
ESR at time of study, mm/1° hour	0.03 (−0.3–0.4)	0.87		
Rheumatoid factor	0.7 (−13–14)	0.92		
ACPA	−6 (−17–6)	0.32		
DAS28-ESR	1 (−3–6)	0.51		
Remission	ref.			
Low disease activity	3 (−11–16)	0.71		
Moderate and high disease activity	−0.2 (−11–11)	0.98		
DAS28-PCR	2 (−3–8)	0.33		
Remission	ref.			
Low disease activity	−0.07 (−13–12)	0.99		
Moderate and high disease activity	1 (−11–13)	0.83		
SDAI	**0.4 (0.2–0.6)**	**0.002**	**0.5 (0.2–0.7)**	**<0.001**
Remission	ref.			
Low disease activity	3 (−14–19)	0.75		
Moderate and high disease activity	5 (−11–20)	0.55		
CDAI	0.3 (−0.2–0.8)	0.25		
Remission	ref.			
Low disease activity	−0.6 (−13–12)	0.92		
Moderate and high disease activity	−0.1 (−12–12)	0.98		
History of extraarticular manifestations	3 (−8–14)	0.59		
Erosions	−5 (−15–5)	0.32		
Current drugs				
Prednisone	0.6 (−9–11)	0.91		
Prednisone doses, mg/day	−0.2 (−3–2)	0.86		
NSAIDs	8 (−2–18)	0.11	7 (−3–17)	0.18
DMARDs	−4 (−19–11)	0.63		
Methotrexate	2 (−8–13)	0.64		
Leflunomide	−6 (−17–6)	0.34		
Anti TNF therapy	−2 (−13–9)	0.73		
Tocilizumab	8 (−19–34)	0.57		
Rituximab	7 (−13–27)	0.51		
Abatacept	−4 (−24–16)	0.69		
Baricitinib	−9 (−43–24)	0.58		
Tofacitinib	−11 (−69–47)	0.72		

In this analysis, 3-NT is the dependent variable. Multivariable analysis is adjusted for age and sex. NSAID: nonsteroidal anti-inflammatory drugs; DMARD: disease-modifying antirheumatic drug; TNF: tumor necrosis factor; ESR: erythrocyte sedimentation rate; DAS28: Disease Activity Score in 28 joints; CRP: C-reactive protein; ACPA: anti-citrullinated protein antibody; CDAI: Clinical Disease Activity Index; SDAI: Simple Disease Activity Index. Significant *p* values are depicted in bold.

## Data Availability

Data will be made available on request.

## Abbreviations

The following abbreviations are used in this manuscript:

3-NT	3-Nitrotyrosine
RA	Rheumatoid arthritis
CV	Cardiovascular
DAS28	Disease Activity Score 28-joint count
ACR	American Colleague of Rheumatology
EULAR	European Alliance of Associations for Rheumatology
CDAI	Clinical Disease Activity Index
SDAI	Simple Disease Activity Index
NCEP	National Cholesterol Education Program
ACPA	anti-citrullinated protein antibody
SCORE2	Systematic Coronary Risk Evaluation-2
HDL	High-density cholesterol
SCORE2-OP	Systematic Coronary Risk Evaluation-2 Older Persons
LDL	Low-density cholesterol
HOMA	Homeostatic model assessment
IR	Insulin resistance
cIMT	Carotid intima-media
SD	Standard deviation
IQR	Interquartile range
BMI	Body mass index
CRP	C-reactive protein
DMARD	Disease-modifying antirheumatic drug
NSAID	Nonsteroidal anti-inflammatory drugs
TNF	Tumor necrosis factor
